# Lung Cancer Associated with Cystic Airspaces: Current Insights into Diagnosis, Pathophysiology, and Treatment Strategies

**DOI:** 10.3390/cancers16233930

**Published:** 2024-11-24

**Authors:** Kun Wang, Xuechun Leng, Hang Yi, Guochao Zhang, Zhongwu Hu, Yousheng Mao

**Affiliations:** 1 Department of Thoracic Surgery, National Cancer Center/National Clinical Research Center for Cancer/Cancer Hospital, Chinese Academy of Medical Sciences and Peking Union Medical College, Beijing 100021, China; 2 Department of Thoracic Surgery, The Affiliated Huaian No.1 People’s Hospital, Nanjing Medical University, Huai’an 223300, China

**Keywords:** lung cancer associated with cystic airspaces (LCCA), radiomics, clinicopathological characteristics, sequencing analysis, prognosis

## Abstract

Lung cancer associated with cystic airspaces (LCCA) is a rare subtype of non-small-cell lung cancer (NSCLC), accounting for 1–4% of cases. LCCA is characterized by the presence of cystic airspaces within or at the periphery of the tumor on imaging. LCCA poses significant clinical challenges due to its high risk of misdiagnosis or missed diagnosis, often leading to a worse prognosis compared to other forms of lung cancer. While previous studies have identified correlations between the pathological features and imaging characteristics of LCCA, research on its associated driver gene mutations and responses to chemotherapy and immunotherapy remains limited. Furthermore, the development of an appropriate T-staging system is necessary to improve prognostic outcomes. This review provides an overview of the current research on the definition, imaging classification, pathological and molecular mechanisms, and prognosis of LCCA, aiming to provide a reference for clinical decision-making.

## Simple Summary

Lung cancer associated with cystic airspaces is a rare subtype of non-small-cell lung cancer characterized by air-filled cystic structures that can often be misdiagnosed due to similarities with benign conditions. This review highlights the imaging characteristics, pathogenesis, and prognostic factors of LCCA. It emphasizes the challenges in accurately diagnosing and staging LCCA due to its unique features and distinct pathological structure. Understanding these complexities is crucial for improving treatment strategies and patient outcomes, highlighting the need for integrated approaches in clinical management.

## 1. Introduction

Lung cancer is the most prevalent cancer worldwide and remains a leading cause of cancer-related mortality [1,2]. According to a 2020 World Health Organization report, there are 2 million new lung cancer cases and approximately 1.8 million lung cancer-related deaths worldwide each year. Non-small-cell lung cancer (NSCLC) is the most prevalent pathological subtype, accounting for approximately 85% of all lung cancer cases [3]. Most NSCLC lesions present as solid or sub-solid, while others present as pure ground-glass nodules on imaging [4,5,6,7,8,9]. Lung cancer associated with cystic airspaces (LCCA) is a subtype of NSCLC characterized by the presence of air-filled cystic structures within the tumor on imaging. This specific subtype was first reported in 1940 [10]. The overall incidence of LCCA is about 1–4% [11,12]. Imaging features of LCCA are often mistaken for bullae or lung cysts, which increases the risk of misdiagnosis. The thin-walled or air-filled cystic nature of LCCA often results in insufficient tissue sampling during CT-guided biopsy. Additionally, LCCA is frequently peripheral, resulting in low positive rates in sputum cytology and bronchoscopy, further complicating diagnosis. In the Nederland-Leuvens Longkanker Screenings Onderzoek (NELSON) lung cancer screening trial, the rate of missed or delayed diagnosis for LCCA was as high as 22.7% [13].

Despite its overall rarity, the incidence of LCCA is increasing due to the widespread use of CT in clinical practice and advancements in lung cancer screening programs [14]. Current lung nodule guidelines primarily focus on solid, part-solid, or ground-glass nodules [15,16], with no specific strategies for managing cystic lung lesions. Furthermore, research on the epidemiological characteristics, biological behavior, and prognosis of LCCA remains limited. This review aims to summarize recent advances related to imaging classification, pathological mechanisms, and prognostic characteristics of LCCA, aiming to identify new research questions and provide direction for future studies.

## 2. Imaging Characteristics and Classification of LCCA

The diagnosis, differential diagnosis, and classification of LCCA are primarily based on imaging features. The international definition of LCCA describes it as a malignant tumor that forms cavities or cysts within the lung parenchyma, typically resulting from tumor necrosis, liquefaction, or gas accumulation [16]. On high-resolution CT (HRCT), LCCA presents as one or more cystic components, often accompanied by ground-glass opacities or consolidative areas adjacent to the cyst walls or interspersed between cystic spaces. Additionally, the surrounding lung tissue may show localized signs of emphysema or fibrosis.

**Figure 1:**
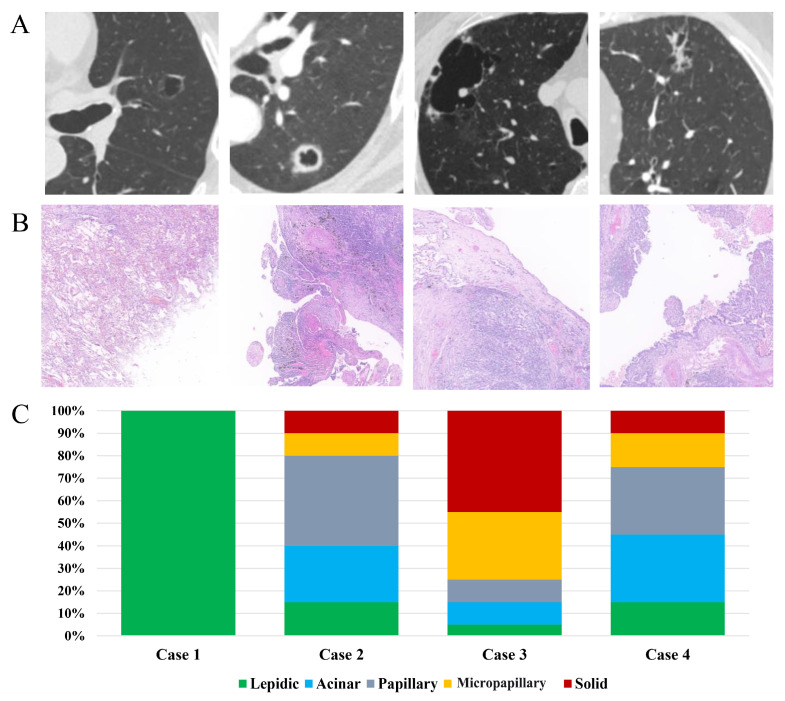
Typical imaging classifications and pathological features of LCCA. (**A**) Four imaging classifications of LCCA. Type I: thin-walled type; Type II: thick-walled type; Type III: mural nodule type; Type IV: mixed type (each from a different patient). (**B**) Postoperative pathological findings of LCCA classifications (H&E staining, 10× magnification). Type I: minimally invasive adenocarcinoma; Type II: poorly differentiated invasive adenocarcinoma; Type III: poorly differentiated invasive adenocarcinoma with vascular invasion; Type IV: moderately differentiated invasive adenocarcinoma. (**C**) Comparison of lung adenocarcinoma subtypes in postoperative LCCA classifications. Type I: minimally invasive adenocarcinoma, predominantly lepidic type; Type II: predominantly papillary-type adenocarcinoma; Type III: predominantly solid-type adenocarcinoma; Type IV: predominantly acinar-type adenocarcinoma.

**Figure 2:**
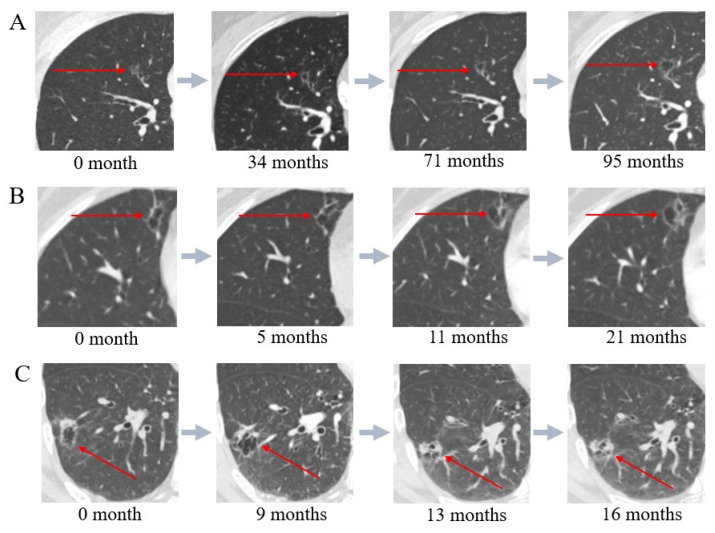
Different progression patterns of LCCA. (**A**) Enlargement of the cystic cavity during follow-up. (**B**) An increase in the number of segmented cystic spaces with the addition of ground-glass opacity components in the cyst wall during follow-up. (**C**) Reduction of the cystic cavity with an increase in solid components in the wall nodules during follow-up. Red arrows indicate lung cancer associated with cystic airspaces.

Currently, there are three primary imaging classifications for LCCA, along with one model of its dynamic progression. Mascalchi et al. categorized LCCA into four types: (1) Type I: extrinsic nodules outside the cyst; (2) Type II: nodules within the cyst; (3) Type III: circumferential thickening of the cyst wall; and (4) Type IV: multiloculated cysts with mixed solid or sub-solid nodules [17]. Shen et al. refined this into four categories (Figure 1): (1) Type I: thin-walled type, with an average wall thickness <2 mm; (2) Type II: thick-walled type, with an average wall thickness ≥ 2 mm; (3) Type III: mural nodule type, featuring internal or external solid nodules; and (4) Type IV: mixed type, with solid or ground-glass components within multiple cysts [18]. Fintelmann et al. developed a more complex classification system consisting of three components: (A) the type of cystic lesion; (B) the consistency of the wall nodules or wall thickening; and (C) the number of internal cystic spaces [19]. Jung et al. proposed a stepwise progression model for LCCA (Figure 2) where stage 1 involves the appearance of cystic spaces within a sub-solid nodule, stage 2 is marked by the growth of these cystic spaces, stage 3 introduces solid components along the edges of the cyst walls, and stage 4 involves the encasement and thickening of the cysts by solid components, eventually leading to cyst contraction [20]. While these classifications detail imaging features, they offer limited prognostic value or guidance for surgical decision-making.

Although PET-CT is widely used for diagnosing and staging lung nodules and cancer, its role in cystic airspace-associated tumors remains less clear. In Mascalchi’s Type III and IV lesions, SUV uptake may be reduced due to the presence of cystic changes interspersed within the consolidated regions [17]. In recent years, advancements in radiomics have provided new insights into the diagnosis and management of LCCA. CT with imaging segmentation techniques can analyze the microstructures of cyst walls and surrounding tissues. By extracting features such as tumor morphology, margin characteristics, texture, and the signal intensity distribution within the cyst wall and internal tissues, more accurate models can be developed for predicting LCCA’s pathological differentiation, identifying high-risk pathological factors, and forecasting postoperative prognosis. Furthermore, dynamic contrast-enhanced magnetic resonance imaging (DCE-MRI) is being explored as a tool to assess LCCA’s vascularization and biological activity. DCE-MRI provides hemodynamic parameters, enabling differentiation between active tumor tissue and inactive areas, such as necrotic or liquefied tissues within the cyst. The hemodynamic parameters obtained using DCE-MRI offer quantifiable insights into the tumor microenvironment, enabling clinicians to assess tumor malignancy more accurately and develop personalized follow-up and treatment plans. However, no studies have yet investigated the use of radiomics and DCE-MRI in LCCA, marking this as a promising area for future research.

## 3. Pathogenesis of LCCA

LCCA can develop in patients with emphysema or pulmonary bullae, but it can also arise in normal lung tissue. Several hypotheses have been proposed to explain the formation of cystic airspaces in LCCA: (1) Tumor cells grow along the alveolar walls, causing the damaged alveolar walls to fuse and form cystic airspaces. Farooqi et al. suggested that limited airflow in the compressed parenchymal and connective tissue regions around pulmonary bullae could lead to the deposition of microorganisms on the bulla walls, resulting in repeated infections. This recurrent inflammation may cause fibrotic scarring around the alveoli, leading to the accumulation of carcinogenic substances [14,21]. (2) Insufficient angiogenesis within the tumor leads to degeneration, liquefaction, and necrosis, with the necrotic tissue being discharged and forming cystic spaces [22]. (3) Elastic retraction of the surrounding lung tissue leads to the formation of airspaces and thinning of the cyst walls [23,24]. (4) The widely accepted “check-valve effect” hypothesis is based on a one-way valve mechanism wherein air accumulates within the cystic airspace, causing it to expand. The subsequent shrinking phase occurs as tumor tissue fills the cyst, and the size of the cystic space does not always correlate with tumor invasion [25].

A CT follow-up study of 89 LCCA cases by Mendoza et al. found that most patients (61/89, 68.5%) showed an increase in wall nodules, while nearly half (43/89, 48.3%) exhibited intermittent thickening of the cyst walls. Complete transformation into a solid mass with the disappearance of the cystic component was rare (11/89, 12.4%), and only a few patients showed no changes in the lesions (4/89, 4.5%) [26].

The relationship between LCCA and emphysema remains unclear. In a retrospective analysis of 123 LCCA patients, Shen et al. found that only 30.9% of the patients had radiological evidence of emphysema. Araki et al. reported that the incidence of emphysema in benign lung cysts was 12.5% (25/200), while this proportion increased to 47–76% in lung cancer patients [27,28]. Further large cohort studies are needed to confirm potential gender differences in LCCA populations and to explore the relationship between LCCA, smoking history, and underlying lung diseases.

## 4. Pathological Characteristics of LCCA

LCCA presents complex and diverse pathological features. Microscopically, LCCA presents as a malignant tumor with cavity or cystic structures, with cyst walls composed of cancer cells exhibiting various growth patterns and cytological features [14]. LCCA is typically classified as NSCLC, with adenocarcinoma being the most common subtype. It was reported in up to 80% and 88% of the cases in the largest cohort studies (the Fintelman and Farooqi cohorts, respectively) [14,19]. However, specific subtypes of lung adenocarcinoma in LCCA have not been extensively studied. The high proportion of poorly differentiated components (such as micropapillary or solid adenocarcinoma) may contribute to the poor prognoses of LCCA patients. Wang et al. proposed that LCCA with multilocular cysts is a marker of adenocarcinoma invasiveness, with the invasiveness of the tumor correlating with the number of cysts (47.1% vs. 72.2%, *p* < 0.05) [25]. Shen further suggested that multicystic LCCA is associated with poorly differentiated adenocarcinoma (odds ratio [OR], 6.5; 95% confidence interval [CI], 1.1–36.4; *p* = 0.035) [18]. Pan et al. found that when the solid component of LCCA exceeded 30%, postoperative pathology often revealed invasive adenocarcinoma. In cases where the solid component was less than 30% and accompanied by well-defined ground-glass opacities, minimally invasive adenocarcinoma was more frequently observed. When CT revealed a solitary thick-walled cystic airspace with or without wall nodules and no peripheral ground-glass opacities, postoperative pathology typically identified squamous cell carcinoma or adenosquamous carcinoma [29]. This suggests that cyst formation may be related to the growth patterns of adenocarcinoma, which are typically well-differentiated and may produce mucus. Squamous cell carcinoma is the second most common subtype and is often associated with thicker cyst walls, especially in smokers.

Follow-up studies of LCCA patients conducted by Jung et al. indicated that LCCA initially manifests as a thin fibrous wall surrounding the cystic space, which thickens as the disease progresses, becoming covered by adenocarcinoma cells. This thickening of the fibrous wall is accompanied by invasive adenocarcinoma cell infiltration and stromal reactions, leading to fibrosis. Patients with late-stage LCCA often present with more advanced TNM staging and intraluminal spread [20,30]. Additionally, vascular invasion is frequently observed in LCCA, with prominent neovascularization and fibrosis of the inner cyst wall serving as characteristic pathological features. This fibrosis differs from typical stromal reactions and precedes the appearance of tumor cells [31]. Pure ground-glass opacities surrounding the cyst are uncommon in LCCA, and once solid components appear, postoperative pathology often reveals invasive characteristics. This suggests a dichotomous pattern in LCCA characterized by rapid tumor invasion. The mechanisms driving this rapid tumor infiltration remain to be explored.

LCCA exhibits a complex tumor microenvironment that includes inflammatory cell infiltration, fibrosis, and necrosis. A deeper understanding of the pathological characteristics of LCCA could help elucidate its mechanisms of growth and invasion and could offer crucial insights for clinical management. The pathological features and classification of LCCA are critical for both diagnosis and treatment. They help guide early therapeutic interventions and provide essential information on patient prognosis [32].

Pathological image segmentation technology, using digital pathology, can accurately separate tumor tissue from complex pathological images. By applying computer vision and machine learning techniques to segment microscopic tumor structures—such as cell morphology, mitotic activity, necrotic areas, and histological characteristics of the cyst walls—the recognition and classification accuracy of LCCA features can be significantly enhanced [33]. This technology not only improves the precision of the pathological diagnosis and classification of LCCA but also opens avenues for further research into the growth patterns, invasiveness, and potential metastatic pathways of LCCA.

## 5. Personalized Treatment for LCCA

Treatment options for LCCA patients include surgery, drug therapy, and radiotherapy. Surgery remains the primary treatment for early-stage LCCA patients and has a good prognosis. However, due to the unique characteristics of LCCA, traditional TNM staging may not accurately guide clinical evaluation, posing challenges for clinicians in selecting optimal surgical timings. The choice of adjuvant therapies often depends on the pathological features and molecular markers of LCCA. Research on the molecular mechanisms of LCCA, particularly the genomic and epigenetic variations that influence tumor progression and treatment responses, remains limited. A meta-analysis by Mendoza et al., which included eight study cohorts and 341 patients, identified the most common driver mutations in LCCA as EGFR (46/122, 37.7%) and KRAS (21/122, 17.2%) [26]. This finding is consistent with research by Guo et al. [34]. Interestingly, Fintelmann et al. reported that KRAS mutations were detected in 64% of their LCCA cohort [19]. The variation in mutation prevalence could be attributed to genetic differences between Asian and Caucasian populations [35,36]. Targeted therapies for LCCA primarily focus on drugs associated with driver gene mutations. Unfortunately, there are no reported data on the clinical and pathological response rates of LCCA patients that received adjuvant therapy.

Currently, there is a paucity of studies evaluating PD-L1 expression levels and the response to immunotherapy in LCCA. Toyokawa et al. demonstrated that among lung adenocarcinoma patients, those with emphysema had higher PD-L1 expression levels compared to those without emphysema [37,38]. They analyzed 257 patients who had received neoadjuvant immunotherapy and found that the NSCLC patients with emphysema had significantly longer progression-free survival (PFS) and overall survival (OS) compared to those without emphysema (*p* = 0.0407 and *p* = 0.0126). It remains uncertain whether LCCA patients exhibit high PD-L1 expression and could benefit from immunotherapy.

Regarding the epigenetic features of LCCA, current research is limited. DNA methylation and histone modifications may influence the growth and invasiveness of tumor cells. Epigenetic changes and driver gene mutations work together to influence the patient’s response to treatment. Future studies should focus on how epigenetic regulation can be harnessed to develop new targeted treatment strategies.

Given the biological characteristics and heterogeneity of LCCA, personalized treatment is particularly crucial. With a deeper understanding of LCCA’s molecular mechanisms, targeted therapies specific to particular mutations and epigenetic features are gradually becoming feasible. By identifying the specific molecular markers of a tumor, a personalized treatment regimen can be designed to improve efficacy and reduce adverse effects. This could provide new directions for the clinical management of LCCA.

## 6. Prognostic Characteristics of LCCA Populations

Research on the prognostic characteristics of LCCA populations is currently scarce. In a retrospective analysis of 123 LCCA patients, Shen et al. found that patients with Type I (thin-walled) LCCA had longer PFS compared to those with non-LCCA lung cancer, whereas patients with Type III (cystic airspaces with mural nodules) LCCA had the poorest PFS [39]. Watanabe et al. reported that patients with thick-walled LCCA had higher rates of postoperative recurrence and distant metastasis compared to those with thin-walled LCCA [40]. Similarly, Ma et al. found that patients with thick-walled LCCA (Shen Type II) had the worst prognosis, while those with thin-walled LCCA (Shen Type I) had more favorable outcomes [41].

Jung’s study demonstrated that the imaging progression stages of LCCA were strongly correlated with prognosis. The 5-year recurrence-free survival (RFS) rate was 100% in stage II but dropped significantly to 56% in stage III. By stage IV, the 5-year RFS further declined to 16%, with an OS of 48%, the lowest among all stages. Additionally, the study found that the thicker the solid cyst wall, the higher the recurrence rate in patients with LCCA. Thickening of the solid cyst wall was associated with an increased volume of invasive components in sub-solid nodules and greater lesion aggressiveness [20].

## 7. Conclusions

One of the defining characteristics of LCCA is the presence of airspaces or cysts within the tumor, which can complicate T-staging assessments. The traditional TNM staging system, which primarily focuses on tumor size, lymph node involvement, and distant metastasis, may not fully apply to LCCA. Cystic structures within the tumor can result in an overestimation of tumor size, and staging based solely on tumor dimensions may fail to accurately reflect the true malignancy or prognosis in LCCA patients. Moreover, due to its distinct pathological structure, LCCA may respond differently to radiotherapy and chemotherapy compared to other solid tumors. Prognosis can vary considerably depending on the specific imaging subtype, highlighting the need for further high-quality research that incorporates LCCA’s imaging characteristics, molecular pathology, and immune microenvironment to address existing knowledge gaps. For LCCA, diagnosis, staging, and treatment strategies are increasingly dependent on integrated multimodal evaluations. Advances in technologies such as imaging segmentation can provide a deeper understanding of LCCA’s complexity, facilitate the optimization of treatment plans, and ultimately improve patient outcomes.

## References

[1] Thai A.A., Solomon B.J., Sequist L.V., Gainor J.F., Heist R.S. (2021). Lung cancer. Lancet.

[2] Ettinger D.S., Wood D.E., Aisner D.L., Akerley W., Bauman J.R., Bharat A., Bruno D.S., Chang J.Y., Chirieac L.R., D’Amico T.A. (2022). Non-Small Cell Lung Cancer, Version 3.2022, NCCN Clinical Practice Guidelines in Oncology. J. Natl. Compr. Cancer Netw..

[3] Zhang Y., Vaccarella S., Morgan E., Li M., Etxeberria J., Chokunonga E., Manraj S.S., Kamate B., Omonisi A., Bray F. (2023). Global variations in lung cancer incidence by histological subtype in 2020: A population-based study. Lancet Oncol..

[4] Digumarthy S.R., Mendoza D.P., Zhang E.W., Lennerz J.K., Heist R.S. (2019). Clinicopathologic and Imaging Features of Non-Small-Cell Lung Cancer with MET Exon 14 Skipping Mutations. Cancers.

[5] Digumarthy S.R., Mendoza D.P., Lin J.J., Chen T., Rooney M.M., Chin E., Sequist L.V., Lennerz J.K., Gainor J.F., Shaw A.T. (2020). Computed tomography imaging features and distribution of metastases in ROS1-rearranged non-small-cell lung cancer. Clin. Lung Cancer.

[6] Digumarthy S.R., Mendoza D.P., Padole A., Chen T., Peterson P.G., Piotrowska Z., Sequist L.V. (2019). Diffuse Lung Metastases in EGFR-Mutant Non-Small Cell Lung Cancer. Cancers.

[7] Mendoza D.P., Lin J.J., Rooney M.M., Chen T., Sequist L.V., Shaw A.T., Digumarthy S.R. (2020). Imaging Features and Metastatic Patterns of Advanced ALK-Rearranged Non-Small Cell Lung Cancer. AJR Am. J. Roentgenol..

[8] Mendoza D.P., Stowell J., Muzikansky A., Shepard J.O., Shaw A.T., Digumarthy S.R. (2019). Computed Tomography Imaging Characteristics of Non-Small-Cell Lung Cancer With Anaplastic Lymphoma Kinase Rearrangements: A Systematic Review and Meta-Analysis. Clin. Lung Cancer.

[9] Mendoza D.P., Dagogo-Jack I., Chen T., Padole A., Shepard J.O., Shaw A.T., Digumarthy S.R. (2019). Imaging characteristics of BRAF-mutant non-small cell lung cancer by functional class. Lung Cancer.

[10] Womack N.A., Graham E.A. (1941). Epithelial metaplasia in congenital cystic disease of the lung: Its possible relation to carcinoma of the bronchus. Am. J. Pathol..

[11] Vlahos I., Stefanidis K., Sheard S., Nair A., Sayer C., Moser J. (2018). Lung cancer screening: Nodule identification and characterization. Transl. Lung Cancer Res..

[12] Tan Y., Gao J., Wu C., Zhao S., Yu J., Zhu R., Zhang Q., Wu G., Xue X., Wu J. (2019). CT Characteristics and Pathologic Basis of Solitary Cystic Lung Cancer. Radiology.

[13] Scholten E.T., Horeweg N., de Koning H.J., Vliegenthart R., Oudkerk M., Mali W.P., de Jong P.A. (2015). Computed tomographic characteristics of interval and post screen carcinomas in lung cancer screening. Eur. Radiol..

[14] Farooqi A.O., Cham M., Zhang L., Beasley M.B., Austin J.H., Miller A., Zulueta J.J., Roberts H., Enser C., Kao S.J. (2012). Lung cancer associated with cystic airspaces. AJR Am. J. Roentgenol..

[15] Adams S.J., Stone E., Baldwin D.R., Vliegenthart R., Lee P., Fintelmann F.J. (2023). Lung cancer screening. Lancet.

[16] Steiling K., Kathuria H., Echieh C.P., Ost D.E., Rivera M.P., Begnaud A., Celedón J.C., Charlot M., Dietrick F., Duma N. (2023). Research Priorities for Interventions to Address Health Disparities in Lung Nodule Management: An Official American Thoracic Society Research Statement. Am. J. Respir. Crit. Care Med..

[17] Mascalchi M., Attinà D., Bertelli E., Falchini M., Vella A., Pegna A.L., Ambrosini V., Zompatori M. (2015). Lung cancer associated with cystic airspaces. J. Comput. Assist. Tomogr..

[18] Shen Y., Xu X., Zhang Y., Li W., Dai J., Jiang S., Wu T., Cai H., Sihoe A., Shi J. (2019). Lung cancers associated with cystic airspaces: CT features and pathologic correlation. Lung Cancer.

[19] Fintelmann F.J., Brinkmann J.K., Jeck W.R., Troschel F.M., Digumarthy S.R., Mino-Kenudson M., Shepard J.O. (2017). Lung Cancers Associated with Cystic Airspaces: Natural History, Pathologic Correlation, and Mutational Analysis. J. Thorac. Imaging.

[20] Jung W., Cho S., Yum S., Chung J.H., Lee K.W., Kim K., Lee C.T., Jheon S. (2020). Stepwise Disease Progression Model of Subsolid Lung Adenocarcinoma with Cystic Airspaces. Ann. Surg. Oncol..

[21] Singh N., Bal A. (2012). Lung cyst caused by centrally located bronchogenic carcinoma. Arch. Bronconeumol..

[22] Lan C.C., Wu H.C., Lee C.H., Huang S.F., Wu Y.K. (2010). Lung cancer with unusual presentation as a thin-walled cyst in a young nonsmoker. J. Thorac. Oncol..

[23] Iwata T., Nishiyama N., Nagano K., Izumi N., Tsukioka T., Hanada S., Kimura T., Kudoh S., Hirata K., Suehiro S. (2009). Squamous cell carcinoma presenting as a solitary growing cyst in lung: A diagnostic pitfall in daily clinical practice. Ann. Thorac. Cardiovasc. Surg..

[24] Zhu H., Zhang L., Huang Z., Chen J., Sun L., Chen Y., Huang G., Chen Q., Yu H. (2023). Lung adenocarcinoma associated with cystic airspaces: Predictive value of CT features in assessing pathologic invasiveness. Eur. J. Radiol..

[25] Wang B., Hamal P., Sun K., Bhuva M.S., Yang Y., Ai Z., Sun X. (2021). Clinical Value and Pathologic Basis of Cystic Airspace Within Subsolid Nodules Confirmed as Lung Adenocarcinomas by Surgery. Clin. Lung Cancer.

[26] Mendoza D.P., Heeger A., Mino-Kenudson M., Lanuti M., Shepard J.O., Sequist L.V., Digumarthy S.R. (2021). Clinicopathologic and Longitudinal Imaging Features of Lung Cancer Associated With Cystic Airspaces: A Systematic Review and Meta-Analysis. AJR Am. J. Roentgenol..

[27] Araki T., Nishino M., Gao W., Dupuis J., Putman R.K., Washko G.R., Hunninghake G.M., O’Connor G.T., Hatabu H. (2015). Pulmonary cysts identified on chest CT: Are they part of aging change or of clinical significance?. Thorax.

[28] Mouronte-Roibás C., Leiro-Fernández V., Fernández-Villar A., Botana-Rial M., Ramos-Hernández C., Ruano-Ravina A. (2016). COPD, emphysema and the onset of lung cancer. A systematic review. Cancer Lett..

[29] Pan X., Wang H., Yu H., Chen Z., Wang Z., Wang L., Chen J. (2020). Lung cancer associated with cystic airspaces: CT and pathological features. Transl. Cancer Res..

[30] Travis W.D., Brambilla E., Noguchi M., Nicholson A.G., Geisinger K.R., Yatabe Y., Beer D.G., Powell C.A., Riely G.J., Van Schil P.E. (2011). International association for the study of lung cancer/american thoracic society/european respiratory society international multidisciplinary classification of lung adenocarcinoma. J. Thorac. Oncol..

[31] Watanabe Y., Kusumoto M., Yoshida A., Suzuki K., Asamura H., Tsuta K. (2015). Surgically resected solitary cavitary lung adenocarcinoma: Association between clinical, pathologic, and radiologic findings and prognosis. Ann. Thorac. Surg..

[32] Snoeckx A., Reyntiens P., Carp L., Spinhoven M.J., El Addouli H., Van Hoyweghen A., Nicolay S., Van Schil P.E., Pauwels P., van Meerbeeck J.P. (2019). Diagnostic and clinical features of lung cancer associated with cystic airspaces. J. Thorac. Dis..

[33] Wu J., Yuan T., Zeng J., Gou F. (2023). A Medically Assisted Model for Precise Segmentation of Osteosarcoma Nuclei on Pathological Images. IEEE J. Biomed. Health Inform..

[34] Guo J., Liang C., Sun Y., Zhou N., Liu Y., Chu X. (2016). Lung cancer presenting as thin-walled cysts: An analysis of 15 cases and review of literature. Asia Pac. J. Clin. Oncol..

[35] Arbour K.C., Jordan E., Kim H.R., Dienstag J., Yu H.A., Sanchez-Vega F., Lito P., Berger M., Solit D.B., Hellmann M. (2018). Effects of Co-Occurring Genomic Alterations on Outcomes in Patients with KRAS-Mutant Non-Small Cell Lung Cancer. Clin. Cancer Res..

[36] Ahn B., Yoon S., Kim D., Chun S.M., Lee G., Kim H.R., Jin Jang S., Sang Hwang H. (2022). Clinicopathologic and genomic features of high-grade pattern and their subclasses in lung adenocarcinoma. Lung Cancer.

[37] Toyokawa G., Takada K., Okamoto T., Kozuma Y., Matsubara T., Haratake N., Takamori S., Akamine T., Katsura M., Shoji F. (2017). High Frequency of Programmed Death-Ligand 1 Expression in Emphysematous Bullae-Associated Lung Adenocarcinomas. Clin. Lung Cancer.

[38] Toyokawa G., Shimokawa M., Kozuma Y., Matsubara T., Haratake N., Takamori S., Akamine T., Takada K., Katsura M., Shoji F. (2018). Invasive features of small-sized lung adenocarcinoma adjoining emphysematous bullae. Eur. J. Cardiothorac. Surg..

[39] Shen Y., Zhang Y., Guo Y., Li W., Huang Y., Wu T., Jiang G., Dai J. (2021). Prognosis of lung cancer associated with cystic airspaces: A propensity score matching analysis. Lung Cancer.

[40] Watanabe Y., Kusumoto M., Yoshida A., Shiraishi K., Suzuki K., Watanabe S.I., Tsuta K. (2016). Cavity Wall Thickness in Solitary Cavitary Lung Adenocarcinomas Is a Prognostic Indicator. Ann. Thorac. Surg..

[41] Ma Z., Wang S., Zhu H., Li Y., Zhang Y. (2022). Comprehensive investigation of lung cancer associated with cystic airspaces: Predictive value of morphology. Eur. J. Cardio-Thorac. Surg..

